# Virulence of newcastle disease virus: what is known so far?

**DOI:** 10.1186/1297-9716-42-122

**Published:** 2011-12-23

**Authors:** Jos CFM Dortmans, Guus Koch, Peter JM Rottier, Ben PH Peeters

**Affiliations:** 1 Central Veterinary Institute of Wageningen UR, PO Box 65, 8200 AB Lelystad, The Netherlands; 2 Virology Division, Department of Infectious Diseases & Immunology, Utrecht University, Faculty of Veterinary Medicine, PO Box 80.163, 3508 TD Utrecht, The Netherlands

## Abstract

In the last decade many studies have been performed on the virulence of Newcastle disease virus (NDV). This is mainly due to the development of reverse genetics systems which made it possible to genetically modify NDV and to investigate the contribution of individual genes and genome regions to its virulence. However, the available information is scattered and a comprehensive overview of the factors and conditions determining NDV virulence is lacking. This review summarises, compares and discusses the available literature and shows that virulence of NDV is a complex trait determined by multiple genetic factors.

## Table of contents

1 Introduction

2 Determination of NDV virulence

3 Newcastle disease virus

4 Viral entry proteins: major virulence determinants

5 Immune evasion and virulence

6 Replication and virulence

7 Non-coding regions

8 Concluding remarks

9 Acknowledgements

10 Competing interests

11 Authors' contributions

12 References

## 1. Introduction

The virulence of a virus is determined by multiple genetic factors. These may involve its tissue or organ tropism, its ability to deal with the host's immune system and/or its efficacy of replication. In the literature the definition of a virulence factor or virulence determinant is not always clear-cut. Thus, many studies concluded that if a genetic mutation in a gene or a complete knock out of the gene function results in an attenuated phenotype, this particular gene or amino acid sequence is a virulence determinant. However, some proteins or protein domains are involved in basic replication processes, making them essential for virus reproduction. Hence, the terms "virulence factor" and "virulence determinant" should be used with care. Our definition of a virulence determinant is: a naturally occurring genetic difference between strains of the same species that is responsible for their difference in virulence. However, nowadays reverse genetics allows the genetic modification of viral genomes almost at will and as a consequence the effects of genetic modifications that have not been encountered in nature can also be studied. Indeed, these techniques have resulted in much more detailed information on the involvement of viral genes and proteins in the virus life cycle and consequently on their contribution to virulence. Therefore, it is arguable whether a particular "artificial" genetic difference still meets the above mentioned definition of a virulence determinant. Similar effects may occur in nature but may be countered by other factors or may be too subtle to be determined by the particular test used to measure differences in virulence.

Newcastle disease (ND) is one of the most important infectious diseases of poultry. It is distributed worldwide and has the potential to cause large economic losses in the poultry industry [1,2]. Its causative agent is Newcastle disease virus (NDV), a virus that is able to infect over 240 species of birds and which spreads primarily through direct contact between infected and healthy birds [3]. The first outbreaks of ND in Java, Indonesia, and Newcastle-upon-Tyne, England, were reported during the mid-1920s [4,5]. Within a few years ND had spread throughout the world and became endemic in many countries [2].

NDV occurs in the field as a variety of strains which differ extensively in the organ systems that they affect and in the severity of the symptoms that they produce in infected birds. Based on the severity of the disease in chickens, NDV has been classified into three pathotypes: lentogenic, mesogenic and velogenic. Lentogenic NDV strains cause subclinical infection with mild respiratory or enteric disease and are considered low-virulent. Mesogenic NDV strains are of intermediate virulence causing respiratory infection with moderate mortality (< 10%), while velogenic NDV strains are highly virulent causing mortality rates up to 100% [6]. Velogenic strains are further classified into viscerotropic velogenic and neurotropic velogenic strains. Viscerotropic velogenic strains produce lethal haemorrhagic lesions in the viscera, whereas neurotropic velogenic strains cause neurological and respiratory disorders [1,7]. The study of virulence and the identification of viral determinants of disease severity is important because an understanding of the mechanisms underlying the outcome of infection may enable a more effective prophylactic or therapeutic approach for viral diseases.

## 2. Determination of NDV virulence

Low virulent NDV strains need the addition of exogenous trypsin to spread from cell to cell and to form syncytia in cell culture monolayers, whereas virulent strains do not [8,9]. Therefore, it was already suggested that the NDV plaque size correlates with virulence [10,11]. However, other studies have shown that the plaque size is highly dependent on the use of certain viral mutants, strains and cell types, and that it cannot be considered as a reliable marker for viral virulence, leaving the study of this crucial biological feature unfortunately to the realm of animal experimentation [12-16].

Useful in vivo tests for the assessment of virulence are the mean death time (MDT) in embryonated chicken eggs [17], the intravenous pathogenicity index (IVPI) in six-week-old chickens [18] and the intracerebral pathogenicity index (ICPI) in one-day-old chickens [19]. Although in most cases the MDT and the IVPI may give a useful indication of virulence, they are considered to be imprecise, particularly when used to assess viruses isolated from hosts other than chickens [18,20,21]. Consequently, these assays are not considered sufficiently reliable for the characterization of NDV isolates in case of a suspected outbreak [19,22].

The generally accepted method to measure the virulence of NDV strains is the ICPI [19], because of its established accuracy and sensitivity [18]. The variation in virulence of different NDV isolates is reflected in the index which ranges from 0.0 (avirulent viruses) to 2.0. (high virulent viruses). Although useful for the sake of defining virus virulence for control purposes, the use of the ICPI for virulence determination for research goals may be criticized, particularly because intracerebral inoculation is obviously an unnatural way of infection. Indeed, in some cases phenotypic differences observed after intracerebral infection were not observed when the natural infection route was used [23-25]. It should also be noted, that some differences may exist in the execution of the test. In some studies non-standard amounts of inoculum were used for the ICPI [26-28]. In these particular studies 10^3 ^plaque forming units (PFU) were used per chicken, which is probably much less than the amount of virus in the inoculum prescribed by the World Organisation for Animal Health (OIE) and EU guidelines that consists of a 1 to 10 dilution of allantoic fluid with an haemagglutination (HA) titre of at least 2^4 ^[19]. Nevertheless, despite these discrepancies the ICPI test in general is a reliable and reproducible test providing a good indication of the relative virulence of different viruses.

## 3. Newcastle disease virus

NDV is a paramyxovirus and viruses from this family are enveloped, non-segmented, negative-sense RNA viruses, which - together with the *Pneumovirinae *- constitute the family of *Paramyxoviridae *[29]. NDV, or avian paramyxovirus type 1 (APMV-1), is classified in the genus *Avulavirus *of the subfamily *Paramyxovirinae *[29]. NDV viruses belong to one serotype and there are two classes [30]. The genome of class I viruses consists of 15 198 nucleotides (nt) and the genome of class II viruses consists of 15 186 or 15 192 nt. [30]. The genome contains six open reading frames (ORF) which encode the nucleoprotein (NP), the phosphoprotein (P), the matrix protein (M), the fusion protein (F), the haemagglutinin-neuraminidase (HN) and the large protein (L). At least one additional, non-structural protein (V) and possibly a second one (W), are generated by RNA editing during P gene transcription [31].

Virus infection is initiated by attachment of the virion to the surface of the target cell. Binding of the viral HN glycoprotein to sialic acid-containing cell surface proteins, which serve as receptors, triggers the F protein-promoted fusion of the viral envelope with the plasma membrane of the host cell through a pH-independent mechanism, similar to other paramyxoviruses [32]. The viral nucleocapsid or ribonucleoprotein complex (RNP) contains the RNA genome encapsidated with NP and associated with the polymerase complex composed of the P and L proteins. After entry, the viral nucleocapsid dissociates from the M protein and is released into the cytoplasm. Subsequently, the polymerase complex transcribes the viral genomic RNA to produce the mRNAs that are required for the synthesis of the viral proteins. Binding of the polymerase complex to the nucleocapsid is mediated by the P protein, whereas the catalytic activities are functions of the L protein [33-37].

The switch from transcription to genome replication takes place when sufficient amounts of viral protein have accumulated. The polymerase complex is responsible for the synthesis of full-length plus-strand antigenomic RNA, which in turn serves as the template for synthesis of minus-strand genomic RNA. Viral nucleocapsids are then assembled by association of NP with the newly formed genomic RNA and with the polymerase complex. All components of the virus particle are transported to the plasma membrane where they are assembled under the direction of the M protein. Virions are released from the cell by a process of budding (reviewed in [38]). Finally, the neuraminidase activity of the HN protein facilitates the detachment of the virus from the cell and removes sialic acid residues from progeny virus particles to prevent self-aggregation [32,39].

The genome of negative-strand RNA viruses is exclusively present in viral particles in the form of the RNP; naked viral RNA is not infectious. However, the development of reverse genetics for negative-strand RNA viruses has allowed the production of infectious virus from cloned cDNAs and has made genetic modification possible (reviewed in [40] and [41]). Currently, reverse genetics systems for NDV are available for the lentogenic strains LaSota [42-44] Hitchner B1 [45] and AV324/96 [46], the mesogenic strains Beaudette C [47] and Anhinga [48] and the velogenic strains Herts/33 [49], ZJ1 [50] and RecP05 [51]. It should be noted that most rescued viruses are less virulent than the parental wild-type virus from which they were derived. This observation might be explained by genomic bottlenecks during the cloning process that result in loss of genomic variability and viral population fitness [52,53]. Nevertheless, the availability of a reverse genetics system for NDV as well as for other viruses has provided essential information and tools to study the molecular mechanism of viral replication and pathogenesis in great detail.

## 4. Viral entry proteins: major virulence determinants

Entry of many enveloped viruses, including NDV, into host cells often requires the activation of viral fusion glycoproteins through cleavage by intracellular or extracellular proteases. It has been shown that viral glycoprotein activation is often mediated by proteases recognizing either monobasic or multi-basic cleavage sites [54]. In the early days, virulence studies were performed after exposing NDV strains to mutagens in order to induce differences in the functionality of certain proteins. As a result, the mutants gained or lost the ability to form plaques in cell culture and had a shorter or extended MDT in embryonated chicken eggs [55,56]. Extensive in vitro studies of Nagai et al. [57,58] showed that in all studied NDV strains, virulence in chickens correlated with cleavage of the F protein of the virus. Cleavage of the precursor glycoprotein F0 into F1 and F2 by host cell proteases is essential for progeny virus to become infective [9,55,58,59]. Lentogenic viruses have a monobasic amino acid motif at the F cleavage site, ^112^G-R/K-Q-G-R↓L^117^, and are cleaved extracellularly by trypsin-like proteases found in the respiratory and intestinal tract. Mesogenic and velogenic strains have a multi-basic amino acid motif at the F cleavage site, ^112^R/G/K-R-Q/K-K/R-R↓F^117 ^and can be cleaved intracellularly by ubiquitous furin-like proteases [58,60,61]. This results in a systemic infection that is often fatal. Thus, viral replication in the animal is dependent on proteolytic activation of the virus, as predicted from the previous studies in cell culture and chick embryos [59]. It could be concluded that the amino acid sequence at the F protein cleavage site is a major determinant of NDV virulence [58,61].

Consistently, studies with recombinant NDVs generated by means of reverse genetics showed that the virulence increased significantly when the cleavage site of a lentogenic strain was converted into that of a velogenic strain [43,62,63]. In these studies the ICPI increased from 0.00-0.01 to 1.12-1.28 (Table 1). Furthermore, the ICPI of velogenic NDV strain ZJ1 could be decreased from 1.89 to 0.13 by changing 3 nucleotides in the genome sequence that specifies the cleavage site [64]. Also a single amino acid change, Q114R, in the cleavage site resulted in a decrease in the ICPI index [65]. However, there are observations indicating that the ICPI does not always correlate with the severity of clinical disease in adult chickens inoculated via a natural route of infection. In one study, for example, 4-week-old chickens were inoculated intraconjunctivally with the recombinant NDV strain NDFLtag, a derivative of the lentogenic LaSota strain, containing a velogenic cleavage site. When compared with the lentogenic strain only a small effect of the mutation on the pathogenesis in chickens was observed [25]. This study and the observation that the ICPI value of viruses of lentogenic origin containing an artificial velogenic cleavage site is not as high as that of the velogenic strains from which the cleavage site was derived [43,62,63] suggests that there must be other factors that contribute to virulence and to the extent of clinical disease.

**Table 1:** Properties of recombinant NDV strains: F protein cleavage site and virulence.

Virus	Parent	Cleavage site	ICPI	IVPI	Reference
**NDFL**	LaSota	GRQGRL	0.00	0.00	[43]
NDFLtag*	LaSota	RRQRRF	1.28	0.76	[43,67]
**NDFL(F)^H^**	LaSota	RRQRRF	1.31	0.41	[49]
**rLaSota**	LaSota	GRQGRL	0.00	0.00	[42]
**rLaSota V.F**.*	LaSota	RRQKRF	1.12	0.00	[62]
**rNDV**	Clone 30	GRQGRL	0.01		[63]
rNDVF1*	Clone 30	RRQKRF	1.28		[63]
**NDV/ZJ1**	ZJ1	RRQKRF	1.88	2.80	[50]
**NDV/ZJ1FM**†	ZJ1	GRQERL	0.13	0.00	[64]
**FL-Herts**	Herts/33	RRQRRF	1.63	2.29	[49]
**rgAV324**	AV324/96	RRKKRF	0.10	0.00	[46]
**FL-Herts(F)^AV^**	Herts/33	RRKKRF	1.56		[46]
**rgAV324(F)^H^**	AV324/96	RRQRRF	0.00		[46]

* lentogenic cleavage site motif modified into velogenic cleavage site motif.

† velogenic cleavage site motif modified into lentogenic cleavage site motif.

Superscript H: F gene originating from strain Herts/33.

Superscript AV: F gene originating from strain AV324/96.

Several studies have shown that the consequences of an infection are not solely determined by the presence of a multi-basic cleavage site motif in the viral fusion protein. A recent study with an avirulent avian paramyxovirus serotype 2 (APMV-2) virus showed that this virus does not require exogenous protease supplementation for growth in cell culture. In addition, recombinant APMV-2 viruses in which the cleavage site was replaced by that of APMV serotypes 1 to 9 gained in cleavability, replication and syncytium formation in infected cells, but remained avirulent for chickens [66]. Also, a recombinant NDV LaSota virus (NDFLtag), containing a velogenic cleavage site motif, showed an increase in virulence after one passage in chicken brain as determined by an increase in ICPI from 1.3 to 1.7, while sequence analysis of the entire F gene did not show any mutations [67]. Furthermore, some pigeon derived NDV strains, the so-called pigeon paramyxovirus type 1 (PPMV-1), cause minimal disease despite their F proteins having a multiple basic amino acid sequence. However, they do have a virulence potential in chickens that can emerge upon serial passages in these animals [68-71]. Sequence analysis of such passaged viruses showed that the F protein sequence had not changed and could thus not explain the increase in virulence [69,71,72]. This observation has been confirmed using an infectious cDNA clone of PPMV-1 strain AV324 to prove that the low virulence for both chickens and pigeons [46,73] is an inherent property of this particular virus and is not due to the virus preparation actually consisting of a mixture of low- and high-virulent variants. Furthermore, replacement of the F gene of a virulent NDV strain by that of a non-virulent PPMV-1 strain and vice versa did not affect the virulence of the recipient viruses (Table 1), indicating that the non-virulent phenotype of the PPMV-1 strain must be determined by other factors [46].

The HN protein is responsible for the attachment of virus particles to sialic acid-containing receptors on cell surfaces and for triggering the fusion activity of the F protein during entry of the virus into the host cell. In addition it acts as a neuraminidase, removing sialic acid from progeny virus particles to prevent viral self-aggregation [32]. Comparison of the nucleotide sequences of NDV HN genes has demonstrated that there are three different HN genotypes resulting in proteins of 571, 577 or 616 aa. The HN protein of 616 aa was detected in some lentogenic strains and appears to be a precursor that needs to be processed into biologically active HN by proteolytic removal of a small glycosylated C-terminal fragment [57,58,74-76]. Because the F and HN proteins are closely associated in the virion membrane and in view of the correlation between proteolytic activation of the F protein and viral virulence, it was suggested that processing of this HN precursor might also affect virulence. However, a study investigating the effect of the length of the HN open reading frame on virulence could not show any correlation [63].

Several reverse genetics studies have addressed the contribution of HN to virulence either by exchanging genes between strains, by mutating the glycosylation sites, or by mutating specific residues. The results of these studies are, however, not always in agreement and are therefore not conclusive as to the contribution of HN to virulence. In one of these experiments, a recombinant LaSota virus containing the HN protein of the mesogenic Beaudette C strain showed a significant increase in virulence, changing the pathotype of the recombinant virus from lentogenic to mesogenic [27]. In contrast, another study with exactly the same recombinants could not confirm the results of the MDT and ICPI tests, as shown in Table 2[24]. However, this study did show a decrease in IVPI caused by the HN protein when comparing rBC(HN)^L ^with its parent rBC and confirmed the suggestion that the HN protein determines tissue tropism [27]. This also confirmed findings of a previous study in which the HN protein of the low virulent LaSota virus was substituted by that of the velogenic strain Herts or by an HN chimera consisting of the stem region of strain Herts HN and the globular head of that of LaSota, or vice versa [49]. Whereas the ICPI of the resulting recombinants, NDFLtag(HN)^H^, NDFLtag(HN)^LH ^and NDFLtag(HN)^HL ^(Table 2), did not differ from the parent strain NDFLtag, these recombinants did show a significant increase in IVPI value, suggesting that both the stem region and globular head of the HN protein are involved in determining virus tropism and virulence. The same conclusion could be drawn from a study in which the same chimeric HN genes were used to replace the HN gene of the virulent Herts strain (Table 2).

**Table 2:** Properties of recombinant NDV strains: HN protein and virulence.

Virus	Parent	ICPI	IVPI	MDT	Reference
**rBC**	Beaudette C	1.58*		62	[27]
**rBC(HN)^L^**	Beaudette C	1.02*		72	[27]
**rBC**	Beaudette C	1.66	2.06	48	[24]
**rBC(HN)^L^**	Beaudette C	1.58	1.27	60	[24]
**rBC(HN)^Y526Q^**	Beaudette C	1.33		98	[79]
**rLaSota**	LaSota	0.00*	0.00†	96	[27]
**rLaSota**	LaSota	0.19	0.00	> 90	[24]
**rLaSota(HN)^BC^**	LaSota	0.75*	0.38†	84	[27]
**rLaSota(HN)^BC^**	LaSota	0.00	0.00	> 90	[24]
**NDFLtag**	LaSota	1.28	0.76		[49]
**NDFLtag(HN)^H^**	LaSota	1.40	1.83		[49]
**NDFLtag(HN)^LH^**	LaSota	1.28	1.52		[49]
**NDFLtag(HN)^HL^**	LaSota	1.31	1.82		[49]
**FL-Herts**	Herts/33	1.63	2.29	59	[49]
**FL-Herts(HN)^L^**	Herts/33	1.45	0.95	75	Dortmans et al., unpublished
**FL-Herts(HN)^LH^**	Herts/33	1.40	1.76	64	Dortmans et al., unpublished
**FL-Herts(HN)^HL^**	Herts/33	1.26	0.07	86	Dortmans et al., unpublished
**rAnh**	Anhinga	0.89		88	[48]
**rAnh(HN)^Tk^**	Anhinga	1.00		84	[48]
**rAnh(HN)^CA^**	Anhinga	0.86		72	[48]

* ICPI inoculum: 10^3 ^PFU virus/chicken.

† IVPI inoculum: 10^3 ^PFU virus/chicken.

Superscript L: HN gene originating from strain LaSota.

Superscript BC: HN gene originating from strain Beaudette C.

Superscript H: HN gene originating from strain Herts/33.

Superscript LH: stem region originating from strain LaSota, globular head originating from strain Herts/33.

Superscript HL: stem region originating from strain Herts/33, globular head originating from strain LaSota.

Superscript Tk: HN gene originating from strain Turkey/92.

Superscript CA: HN gene originating from strain California/02.

A similar approach was used by Estevez et al. who created chimeras by exchanging the HN gene of a mesogenic strain by that of a neurotropic or viscerotropic velogenic virus [48]. Introduction of such an HN gene in the NDV Anhinga backbone failed to increase virulence from a mesogenic to a velogenic pathotype when introduced via the ocular infection route in both day-old and 4-week-old chickens [77]. Both studies suggested that NDV virulence is determined multigenically.

In a recent study the role was investigated of the F and HN proteins of two phenotypically contrasting viruses: the moderately virulent NDV virus Beaudette C and the avirulent virus APMV-2 [78]. By simultaneously exchanging the F and HN ectodomains between these viruses it was shown that the two contrasting phenotypes correlated with the origin of the F and HN ectodomains when analysed for replication in vitro, syncytium formation, MDT, ICPI, and replication and tropism in 1-day-old and 2-week-old chickens. The authors concluded that these ectodomains together determine cell fusion, tropism, and virulence phenotypes of NDV and APMV-2. Furthermore, the regions of HN that are critical for the species-specific phenotypes include the cytoplasmic tail and stalk domain, but not the globular head domain.

A key amino acid that showed its importance for the biological activity of the HN protein is residue Y526, which is near the sialic acid binding site in the globular head region. Mutation Y526Q resulted in a decrease in viral haemadsorption activity, neuraminidase activity and fusion activity. Furthermore, this mutation had an attenuating effect on the growth kinetics in cell culture, mean death time and the ICPI value [79]. On the other hand, a recent report showed that although a single amino acid substitution I192M, affects both fusion and neuraminidase activity, it had no effect on the virus pathotype [80].

In many cases proper glycosylation of viral proteins is important for their correct function in the virus life cycle [81]. Modification of HN's N-linked glycosylation sites has been shown to decrease NDV virulence [28]. Since most of the glycosylation sites in the NDV HN protein are well conserved, they seem to play an important role in the biological function of the protein.

## 5. Immune evasion and virulence

The interferon (IFN) system is the first line of host defence against virus infection. Interferons induce an antiviral state that can inhibit virus replication and control virus spread. Paramyxoviruses have evolved mechanisms to escape or prevent both IFN production and IFN responsive signal transduction to evade or antagonize the innate immune response of its host [82-84]. Many paramyxovirus IFN evasion activities are mediated by the virus-encoded V protein, which is conserved in the genera of the *Paramyxovirinae*, with the exception of human parainfluenza virus type 1 (HPIV-1), which lacks an intact V ORF [85]. The NDV V protein is derived from the polycistronic P gene, which is edited during transcription by inserting one or two G residues at the conserved editing locus (AAAAAGGG), thereby generating altogether three P-gene-derived mRNA species. The mRNAs encode the open reading frame (ORF) of P (unedited), the V ORF (+1 frameshift), and the W ORF (+2 frameshift) [31,86]. The IFN-antagonist activity of NDV and many other paramyxoviruses has been mapped to the cysteine-rich C-terminal domain of the V protein [26,87,88], which is highly conserved among several paramyxoviruses [82-84].

A recent study showed that the V protein of the mesogenic Beaudette C strain exhibits a greater antagonistic effect on IFN induction in vitro than that of the lentogenic LaSota strain. This might correlate with their different virulence properties in vivo [89]. Previous studies using recombinant NDV that lacked the expression of the V protein already suggested that this protein plays an important role in virulence (Table 3). Its contribution to NDV virulence was demonstrated in chickens [24,26] and embryonated chicken eggs [90,91]. Furthermore, its function was investigated in cell culture [26,87,90,91]. NDV mutants that completely or partially lack the V protein or that contain a mutated V protein show severe growth impairment in vitro, while their replication in embryonated chicken eggs is age-dependent [26,90,91]. Probably, a reduction in growth of mutant viruses in older embryonated eggs is due to maturation of the host innate immune system and, most likely, the inability of the mutant viruses to counteract the IFN response, which is better developed in older embryos [92].

**Table 3:** Properties of recombinant NDV strains: V protein and virulence.

Virus	Parent	ICPI	IVPI	MDT	Reference
**rBC**	Beaudette C	1.58*	1.45†	62	[26]
**rBC/V-stop**	Beaudette C	0.68*	0.00†	96	[26]
**rBC/Edit**	Beaudette C	0.72*	0.00†	98	[26]
**rBC**	Beaudette C	1.66	2.06	48	[24]
**rBC/V-stop**	Beaudette C	1.54	1.07	63	[24]
**rBC/Edit**	Beaudette C	1.19	0.79	80	[24]

* ICPI inoculum: 10^3 ^PFU virus/chicken.

† IVPI inoculum: 10^3 ^PFU virus/chicken.

V-stop: stop codon introduced in the V reading frame; silent to P reading frame.

Edit: disruption of P gene mRNA editing; inhibition of both V and W protein expression.

It has been shown that viruses with mutated V proteins, in contrast to their parent viruses, are unable to degrade the STAT1 protein [26], which is an important element of the interferon signalling pathway [93]. Furthermore, in vitro experiments have shown that these viruses have an increased rate of apoptosis [91]. In addition, in vivo studies showed that the apoptotic rates corresponded to the severity of the clinicopathological disease of different strains [94]. The results obtained in studies on the V protein of NDV are in agreement with in vivo studies of measles virus and Sendai virus pathogenesis, which showed that deletion of the V protein attenuates viral virulence [95,96].

No studies have been published in which the V genes have been exchanged between different NDV strains, as has for instance been done for the F, HN, or L genes. In an unpublished study, the V proteins of the low virulent LaSota virus and the highly virulent Herts virus were compared by expressing them in a V-negative Herts backbone with an intact P ORF. The V genes were inserted between the P and M ORFs. No differences in ICPI value between the two recombinants were observed (M. Tacken, O. de Leeuw and L. Cornelissen, personal communication). Altogether, the studies mentioned clearly indicate that the V protein is essential for survival and replication of the virus in its host. However, more studies, especially using the natural infection route, must be performed to examine whether the V protein is also involved in determining differences in virulence between NDV strains.

## 6. Replication and virulence

A correlation between virulence and the efficiency of viral replication has been observed for many viruses. Although not fully understood, it is quite conceivable that higher levels of viral replication result in more virus production which may overwhelm the host immune response, hence causing enhanced pathogenesis. It has been reported for NDV that reduced levels of RNA synthesis are associated with reduced virulence [97]. For several other paramyxoviruses such as measles virus [98,99], respiratory syncytial virus and parainfluenza virus [100,101], virus attenuation has been associated with mutations in components of the replication complex. For influenza virus the viral polymerase proteins PB2 and PA are known to contribute to virulence [102-105]. In addition, transcriptional and translation control signals may also modulate virulence by controlling protein expression as has, for instance, been described for vesicular stomatitis virus, measles virus, canine distemper virus and NDV [12,106-111].

The involvement of the NP, P and L replication proteins in NDV virulence has been examined by generating chimeric viruses in which genes were exchanged between strains of different pathotypes [13,112]. In one of these studies a recombinant Beaudette C virus containing the L gene of strain LaSota was shown to replicate to higher levels both in vitro and in vivo and to be slightly more virulent than its parental virus in one-day-old chickens (Table 4). However, no effect was found for the NP and P proteins [112]. In contrast, in our own study all three proteins that make up the viral replication complex (NP, P and L) were found to play a significant role in determining the virulence of NDV [13]. By simultaneously exchanging all genes for the replication proteins between strains Herts and AV324, the virulent Herts virus became significantly attenuated, whereas the low virulent AV324 became much more virulent (Table 4). This was also consistent with in vitro studies, which showed that the replication proteins of Herts are more active than those of AV324. However, the role of the individual replication proteins remained less conclusive. One possible explanation is that the individual Herts replication proteins are inherently more active but that optimal activity is dependent on the presence of the cognate interaction partners. This feature has also been previously observed for the recovered influenza 1918 virus, where the gene segments show a synergistic effect [113]. Another possible explanation might be that the proteins of the two investigated viruses originate from two distinct phylogenetic lineages [114] that are less compatible.

**Table 4:** Properties of recombinant NDV strains: NP, P and L proteins and virulence.

Virus	Parent	ICPI	IVPI	MDT	Reference
**rBC**	Beaudette C	1.47*	2.03*	61*	[112]
**rBC(NP)^L^**	Beaudette C	1.31		61*	[112]
**rBC(P)^L^**	Beaudette C	1.24		62*	[112]
**rBC(NPP)^L^**	Beaudette C	1.44		60*	[112]
**rBC(L)^L^**	Beaudette C	1.75*	2.30*	55*	[112]
**rLaSota**	LaSota	0.00*	0.00*	108*	[112]
**rLaSota(NPP)^BC^**	LaSota	0.00*	0.00*	110*	[112]
**rLaSota(L)^BC^**	LaSota	0.00*	0.00*	115*	[112]
**FL-Herts**	Herts/33	1.54			[13]
**FL-Herts(NP)^AV^**	Herts/33	1.35			[13]
**FL-Herts(P)^AV^**	Herts/33	1.33			[13]
**FL-Herts(NPP)^AV^**	Herts/33	1.35			[13]
**FL-Herts(L)^AV^**	Herts/33	1.30			[13]
**FL-Herts(NPPL)^AV^**	Herts/33	0.55			[13]
**rgAV324**	AV324/96	0.10			[13]
**rgAV324(NP)^H^**	AV324/96	0.04			[13]
**rgAV324(P)^H^**	AV324/96	0.25			[13]
**rgAV324(NPP)^H^**	AV324/96	0.70			[13]
**rgAV324(L)^H^**	AV324/96	0.48			[13]
**rgAV324(NPPL)^H^**	AV324/96	1.03			[13]

* value is the average of two independent tests.

Superscript L: gene originating from strain LaSota.

Superscript BC: gene originating from strain Beaudette C.

Superscript AV: gene originating from strain AV324/96.

Superscript H: gene originating from strain Herts/33.

We studied the adaptation of a PPMV-1 strain to chickens during brain passage. After five passages 3 mutations became dominant in the virus population, two in the L protein (N1564S and V1694E) and one in the P protein (N37D) [115]. These mutations resulted in more efficient replication both in vitro and in vivo, indicating that virulence of PPMV-1 for chickens is directly related to the efficiency of virus replication. Further investigations will be needed to unravel the exact mechanisms of viral transcription, replication and host-interaction and their effect on virulence.

The viral matrix (M) protein has been identified as another regulator involved in viral replication. While the M protein is considered to be the central organizer of viral morphogenesis and budding [32], it has also been found to interact with the replication complex and to thereby affect viral transcription and/or replication, as has been shown for rhabdoviruses and some paramyxoviruses [116-121]. With NP as its most likely binding partner [120,122], the M protein associates with the nucleocapsid [116-118]. Upon viral entry of the target cell, the nucleocapsid dissociates from the M protein and is released into the cytoplasm where transcription can occur. The interaction of M with the nucleocapsid might affect transcription and consequently may have an effect on replication. Furthermore, it is known that the M protein traffics between the cytoplasm and the nucleus during the viral infection cycle [38,123]. It has been suggested that the presence of M in the nucleus may result in the inhibition of host cell functions [124,125], although this has not yet been confirmed for NDV. Replacement of the M protein in strain Herts by that of strain AV324 significantly decreased the ICPI value, however, the opposite was not the case, as shown in Table 5[13].

**Table 5:** Properties of recombinant NDV strains: M protein and virulence.

Virus	Parent	ICPI	Reference
**FL-Herts**	Herts/33	1.54	[13]
**FL-Herts(M)^AV^**	Herts/33	1.18	[13]
**rgAV324**	AV324/96	0.10	[13]
**rgAV324(M)^H^**	AV324/96	0.00	[13]

Superscript AV: M gene originating from strain AV324/96.

Superscript H: M gene originating from strain Herts/33.

## 7. Non-coding regions

Untranslated regions (UTRs) in viruses have been shown to play a role in the regulation of viral transcription and translation. In measles virus and canine distemper virus, the long 3'UTR of M and 5'UTR of F play important roles in replication and virulence of the virus [111,126]. A 6 nt insert in the 5'UTR of the NP gene divides the NDV class II viruses into two groups, namely one with a genome length of 15 186 nt and another with a genome length of 15 192 nt [30]. The fact that both groups harbour pathogenic viruses suggests that this 6 nt insert does not play a major role in virulence.

Deletion of the entire 5'UTR of the HN gene was shown to affect transcription and translation of the HN mRNA and thereby virulence [109]. Furthermore, each UTR seems to be specific for a particular position on the genome and for its associated gene [108].

The sizes of the intergenic sequences (IGSs), i.e. the number of nucleotides between the transcription end-box and transcription start-box, of consecutive genes of NDV vary from a single nt for the first three gene boundaries to 31 nt for the F-HN and 47 nt for the HN-L boundaries, respectively. A study has shown that although NDV can tolerate an IGS length of at least 365 nt, the extended lengths of IGSs down-regulated the transcription of the downstream gene. Furthermore, all viruses that had extended or decreased IGS lengths were attenuated in embryonated chicken eggs, day-old chicks and 6-week-old chickens [12]. While it is clear that the above described genetic modifications have an effect on virulence, it is questionable whether the genomic regions and sequences involved also play a role in determining differences in virulence between naturally occurring NDV strains. The sizes of the IGSs and UTRs of NDV are conserved whereas the nucleotide sequence of these regions may vary to some extent, which is not unexpected for non-coding regions.

## 8. Concluding remarks

**Figure 1:**
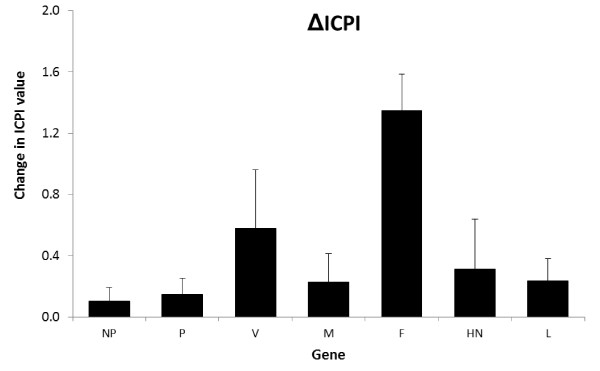
**Summary of the contribution of individual NDV proteins to virulence based on a compilation of the data shown in Tables 1 to 5**. The error bars show standard deviations.

More and more studies illustrate that virulence is a complex trait that is determined by multiple genetic factors. For NDV, the multi-basic amino acid cleavage motif in the F protein is an absolute prerequisite for virulence. In addition, other factors are critically involved in determining virulence, as shown in this review and summarised in Figure 1. However, we must note that the contribution of these factors to virulence may be dependent on the particular virus strain used. For instance, the use of the atypical PPMV-1 strain AV324 has revealed significant effects of the viral replication machinery on virulence. These effects would have been much more difficult to show when using a velogenic strain in which the contribution of the replication complex is probably relatively low compared to that of the F protein cleavage site. Another point to consider is the use of the ICPI as a readout parameter of virulence. As argued above, this test uses an artificial inoculation route and as a consequence it may be questioned whether the results obtained with this test are always relevant for the natural situation. Although this test is often compulsory to determine whether an isolate is virulent or not, its sensitivity may be insufficient to detect subtle differences in virulence.

Many questions still remain concerning the molecular mechanisms used by NDV to regulate tropism, transcription and translation, interference with the host defence machinery, and how they affect virulence. Nevertheless, the studies summarised in this review have contributed significantly to our understanding of the biology of NDV and can serve as an important guide for future research on the molecular principles that determine its virulence.

## 9. Acknowledgements

The authors would like to thank MGJ Tacken, OS de Leeuw and LA Cornelissen for sharing their unpublished data.

## 10. Competing interests

The authors declare that they have no competing interests.

## 11. Authors' contributions

JD developed the structural design of the review and drafted the manuscript. GK and PR reviewed the manuscript. BP supervised the study, and reviewed the manuscript. All authors read and approved the final manuscript.
